# 

**Published:** 1985-07

**Authors:** 


Contents

61 Editorial

62 Animals and Medicine
I. A. Silver

68 Care of the Elderly—The Way Ahead
G. K. Wilcock

72 Vocational Training for General Practice
Michael Lennard

74 The Doctor and the Snake
Philip Radford

77 From our Correspondents

80 From our Foreign Correspondent

82 Obituary—Dr. G. L. Foss

83 Obituary—Dr. O. L. Lloyd

84 Bristol Medico—Chirurgical Society Summer Meeting

86 Exhibition of Art and Craft Work by Bristol Doctors

88 Book Reviews

90 Letter to the Editor

